# Metabolic regulation of ferroptosis in the tumor microenvironment

**DOI:** 10.1016/j.jbc.2022.101617

**Published:** 2022-01-21

**Authors:** Nneka E. Mbah, Costas A. Lyssiotis

**Affiliations:** 1 Department of Molecular & Integrative Physiology, University of Michigan, Ann Arbor, Michigan, USA; 2 Division of Gastroenterology and Hepatology, Department of Internal Medicine, University of Michigan, Ann Arbor, Michigan, USA; 3 Rogel Cancer Center, University of Michigan, Ann Arbor, Michigan, USA

**Keywords:** ferroptosis, cancer, tumor microenvironment, metabolism, tumor immunity, 4-HNE, 4-Hydroxynonenal, 8-OHG, 8-hyroxyguanosine, αKG, alpha ketoglutarate, ACC, Acetyl-CoA carboxylase, ACSL3, acyl-CoA synthetase long-chain family member 3, ACSL4, acyl-CoA synthetase long-chain family member 4, AGER, advanced glycosylation end-product specific receptor, AMPK, 5′ adenosine monophosphate-activated protein kinase, ATM, ataxia-telangiectasia mutated, ATP, adenosine triphosphate, BH2, dihydrobiopterin, BH4, tetrahydrobiopterin, CAF, cancer associated fibroblasts, CD36, cluster of differentiation 36, CHAC1, ChaC Glutathione Specific Gamma-Glutamylcyclotransferase 1, CoA, coenzyme A, CoQ_10_, Coenzyme Q10, CoQ_10_H_2_, Coenzyme Q10 or ubiquinol, CPX, ciclopirox, CTLA4, cytotoxic T-lymphocyte-associated protein 4, DFO, deferoxamine, DHFR, dihydrofolate reductase, DHODH, dihydroorotate dehydrogenase, ePL-PUFA, ether phospholipid-polyunsaturated fatty acids, ETC, electron transport chain, FSP1, ferroptosis suppressor protein1, GCH1, GTP cyclohydrolase 1, GOT1, glutamate oxaloacetate transaminase 1, GPX4, glutathione peroxidase 4, GSH, glutathione, GSSG, glutathione (oxidized), HCAR1, Hydroxycarboxylic acid receptor 1, HMGB1, high mobility group box protein 1, IFNγ, interferon gamma, IFNR, interferon receptor, IKE, imidazole ketone erastin, JAK, Janus kinase, KEAP1, Kelch-like ECH-associated protein 1, LIP, labile iron pool, LOXs, lipoxygenases, LPCAT3, lysophosphatidylcholine acyltransferase 3, MCT1, monocarboxylate transporter 1, MDA, Malondialdehyde, ML162, molecular libraries 162, MUFA, monounsaturated fatty acid, MUFA-PL, monounsaturated fatty acid phospholipid, NRF2, nuclear factor erythroid 2-related factor 2, oxLDL, oxidized low density lipoprotein, OXPHOS, oxidative phosphorylation, P, Phosphate, PD-L1, Programmed death-ligand 1, PLOO, phospholipid hydroperoxyl radical, PLOOH, phospholipid hydroperoxide, PTGS2, Prostaglandin-Endoperoxide Synthase 2, PUFA, polyunsaturated fatty acid, PUFA-PLs, polyunsaturated fatty acid phospholipids, ROS, reactive oxygen species, RSL3, Ras selective lethal 3, SAPE-OOH, oxidized phospholipid, 1-steaoryl-2-15-HpETE-sn-glycero-3-phosphatidylethanolamine, SCD1, stearoyl-CoA desaturase enzyme 1, Se, Selenium, STAT1, signal transducer and activator of transcription 1, STING1, stimulator of interferon genes 1, TAZ, transcriptional co-activator with PDZ-binding motif, TCA, tricarboxylic acid cycle, TLR2, toll-like receptor 2, TME, tumor microenvironment, Ub, Ubiquitin, Vit E, Vitamin E, YAP, Yes-associated protein 1

## Abstract

Ferroptosis is an iron-dependent, nonapoptotic form of regulated cell death triggered by impaired redox and antioxidant machinery and propagated by the accumulation of toxic lipid peroxides. A compendium of experimental studies suggests that ferroptosis is tumor-suppressive. Sensitivity or resistance to ferroptosis can be regulated by cell-autonomous and non-cell-autonomous metabolic mechanisms. This includes a role for ferroptosis that extends beyond the tumor cells themselves, mediated by components of the tumor microenvironment, including T cells and other immune cells. Herein, we review the intrinsic and extrinsic factors that promote the sensitivity of cancer cells to ferroptosis and conclude by describing approaches to harness the full utility of ferroptotic agents as therapeutic options for cancer therapy.

Cancer cells are subjected to and robustly adapt to more oxidative stress than nonmalignant cells (1). Higher oxidative stress in tumors is thought to be caused by altered mitochondrial function and increased activity of reactive oxygen species (ROS)-generating enzymes such as cyclooxygenases, lipoxygenases, and NADPH oxidases, most of which are modulated by tumor-intrinsic factors including increased growth factor and oncogenic signaling (*e.g.*, Ras signaling) and loss of tumor suppressor function (*e.g.*, p53). In addition, external factors such as chemotherapy and radiation also contribute to redox stress in cancer cells. However, if not carefully controlled, unabated ROS can damage macromolecular structures, including proteins and membrane lipids, which can result in cell death or senescence. Therefore, cancer cells rely on endogenous antioxidant networks to maintain redox homeostasis (2, 3, 4).

Lipid ROS is a form of ROS generated by biochemical reactions between oxidant radicals and membrane-lipid polyunsaturated fatty acids (5). This results in oxidative lipid damage that can lead to cell death by ferroptosis. Ferroptosis is a term that describes a nonapoptotic form of cell death associated with the perturbation of redox and antioxidant mechanisms, and propagation of lipid peroxidation reactions. This cell death requires labile active iron and is morphologically, phenotypically, and biochemically distinct from other cell death programs such as apoptosis, necroptosis, pyroptosis, and necrosis (6, 7, 8, 9). Dysregulated metabolism is at the core of ferroptosis, characterized by three hallmarks: (i) impaired antioxidant machinery, (ii) availability of redox-active iron, and (iii) the propagation of toxic lipid hydroperoxides (10).

The importance of ferroptosis in cancer is indicated by its tumor-inhibitory capacity in both primary and drug-resistant cancer cells (11, 12). Much of the now classic work on ferroptosis in cancer has focused on cell-autonomous effects in the tissue culture setting. These studies put forth the idea that ferroptosis may serve a tumor suppressor mechanism, akin to apoptosis or senescence. Contemporary studies have begun to test this idea in animal models, studying cancer ferroptosis and how this is impacted by the tumor microenvironment (TME). The TME contains a myriad of distinct cell types and factors including the tumor cells, stromal cells (*e.g.*, cancer associated fibroblasts), immune cells, vasculature, extracellular matrix (*e.g.*, collagen, fibronectin, laminin), and secreted molecules (*e.g.*, metabolites, cytokines). This collective milieu influences all aspects of tumor biology. For example, oxidative stress is among the many factors influenced by the TME, and this cross talk promotes cancer cell proliferation and survival, cell migration and invasion, wound healing, tumor vascularization defects, and treatment resistance (4, 13). However, there is a delicate balance relating to the impact of ROS in the TME, wherein moderate levels promote tumor growth, while higher levels tilt the balance to increased oxidative damage to macromolecules and cell death (14). In line with this concept, the production of lipid ROS and, by extension the initiation of ferroptosis, could have multifaceted and complex impact on the TME, tumor growth, and therapeutic response.

In this review, we start by setting the stage for ferroptosis as a metabolic form of cell death. We describe early studies that elucidated the mechanisms that form the basis of our understanding of cell-autonomous ferroptosis as tumor-suppressive. Further, we discuss recent studies describing the impact of ferroptosis on the TME. We round out the review by highlighting the therapeutic utility and current challenges mitigating the use of classic ferroptotic agents in preclinical and clinical setting and close by outlining perspectives for future investigation.

## Ferroptosis: When lipid ROS formation outpaces removal

**Figure 1:**
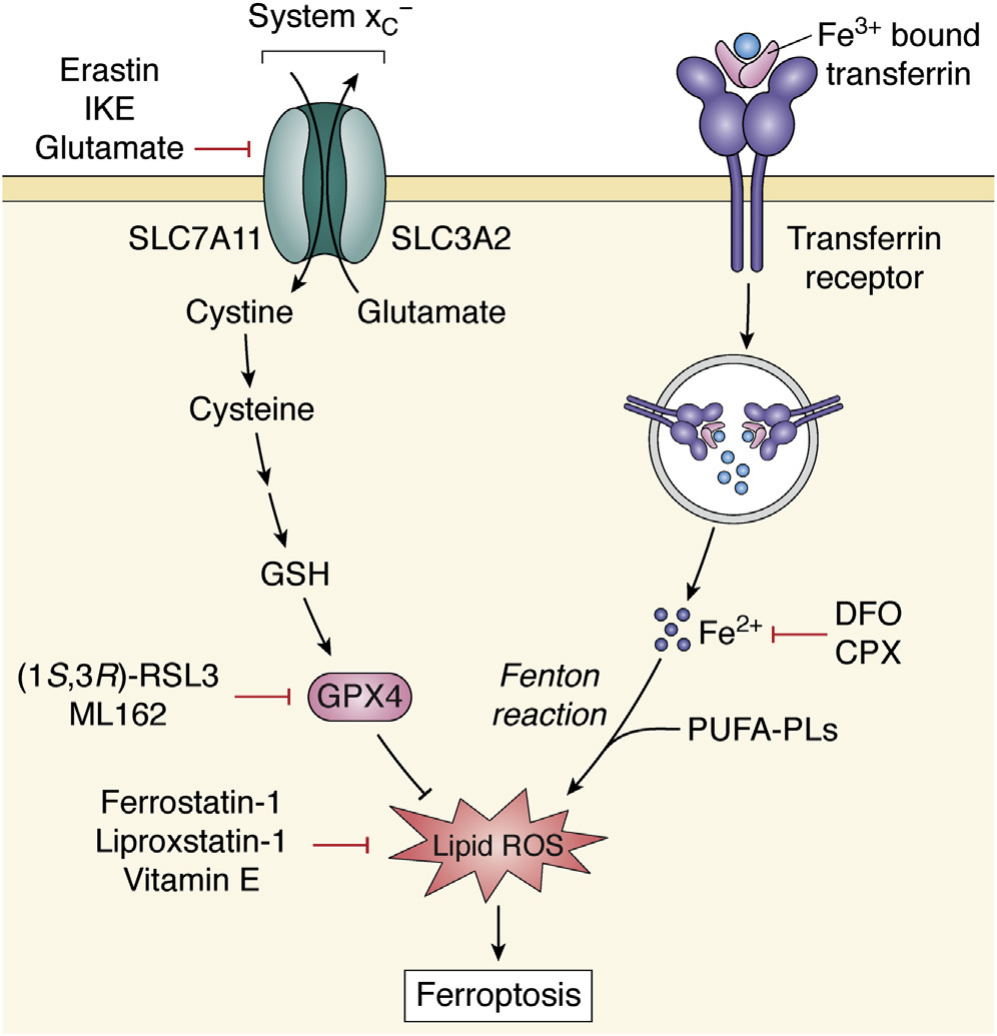
**The first and well-characterized pathway governing ferroptosis.** Ferroptosis is mediated by an iron-dependent accumulation and propagation of lipid peroxidation reactions. Iron (Fe^3+^) bound to transferrin is internalized into cells *via* receptor-mediated endocytosis and subsequently released as redox-reactive iron (Fe^2+^) required for Fenton chemistry. Upstream ferroptosis induction can occur as a result of cysteine starvation *via* inhibition of system x_C_^−^ using small-molecule inhibitors (Erastin, IKE, Glutamate), genetic deletion of *SLC7A11*, or cystine withdrawal, which result in diminished GSH levels, and impaired GPX4 activity. In addition, direct inhibition of GPX4 with small-molecule compounds ((1*S*,3*R*)-RSL3, ML162) or GPX4 depleting class of ferroptosis compounds, can impair antioxidant system and result in the accumulation of lipid ROS leading to ferroptosis. Accordingly, ferroptosis is blocked by iron chelators, DFO and CPX, as well as radical trapping antioxidants, Ferrostatin-1, Liproxstatin-1, and Vitamin E. CPX, ciclopirox; DFO, deferoxamine; GPX4, glutathione peroxidase 4; GSH, glutathione; IKE, imidazole ketone erastin; ML162, molecular libraries 162; PUFA-PLs, polyunsaturated fatty acid phospholipids; ROS, reactive oxygen species; RSL3, Ras selective lethal 3.

Ferroptosis occurs when lipid ROS production exceeds the capacity of the lipid ROS detoxification machinery. Lipid ROS is generated by redox active labile iron *via* Fenton chemistry, and the availability of iron has been shown to be crucial to the initiation of ferroptosis (6) (Fig. 1). The lipid ROS are then spontaneously propagated across unsaturated lipids in the membrane. Specifically, peroxidation of membrane polyunsaturated fatty acid (PUFA) phospholipids (PUFA-PLs) drives the ferroptosis process (15). Studies have identified phosphatidylethanolamine (PE)-containing arachidonic acid (AA; C20:4) and adrenic acid (AdA; C22:4) phospholipids as key substrates of phospholipid peroxidation in ferroptosis (16). The presence of highly oxidizable methylene double bonds makes them major substrates for phospholipid peroxidation (17). The direct connection between accumulation of lipid peroxides and cell death manifests in the fact that ferroptosis can be effectively inhibited by lipophilic antioxidants such as alpha-tocopherol (vitamin E) and its analogs, ferrostatin-1, and liproxstatin-1, all of which serve as radical trapping antioxidants that neutralize lipid ROS and therefore prevent cell death (18, 19).

Detoxification of cellular lipid ROS, which consequently inhibits ferroptosis, can occur through multiple pathways. In fact, many of these pathways have been illuminated by observing that their inhibition promotes ferroptotic cell death. For example, a small-molecule screen for compounds selectively lethal in oncogenic Ras-expressing cells identified what is now considered to be a classic inducer of ferroptosis: *i.e.*, Erastin, a system x_C_^−^ inhibitor (20). System x_C_^−^ is a heterodimeric cystine-glutamate antiporter encoded by *SLC3A2* (CD98 subunit) and the transporter-specific gene *SLC7A11* (xCT subunit) (21, 22). Cystine is the dimeric and oxidized form of cysteine, a nonessential amino acid and the rate-limiting component of glutathione (GSH). GSH is an antioxidant tripeptide and a primary mediator of cellular redox balance. Extracellular cysteine in serum (and tissue culture media) is predominantly present as cystine, and cancer cells employ a variety of mechanisms to increase levels of xCT to obtain cysteine (23). This provides the rationale as to why cancer cells are more vulnerable to ferroptosis initiated by system x_C_^−^ inhibition.

Shortly after the description of system x_C_^−^ inhibitors (24), a second class of ferroptosis-inducing compounds were described that inhibit glutathione peroxidase 4 (GPX4) (20). GPX4 is a GSH-dependent and selenocysteine-containing lipid peroxidase, in which direct or indirect inhibition, *e.g.*, GSH depletion, leads to ferroptosis (20, 25). A notable inhibitor of GPX4 is the small-molecule compound, (1*S*,3*R*)-Ras Selective Lethal 3, abbreviated as RSL3. RSL3 acts by binding covalently to GPX4 and inducing the latter’s inactivation and consequent accumulation of lipid ROS in the cell (25). Thus, these two nodes (system x_C_^−^ and GPX4) converge to promote ferroptosis by way of cysteine depletion and GPX4 inhibition. More recently, other mechanisms have been uncovered that arise from genetic, transcriptional, or metabolic dysregulation that predict sensitivity or resistance to ferroptosis. Some of these directly act on the cysteine-GSH-GPX4 axis, and others work entirely in parallel. In addition, there is now a growing appreciation that the mechanisms regulating ferroptosis in isolated cancer cells in culture are not readily recapitulated *in vivo*. Put another way, the *in vivo* tumor microenvironment can protect cancer cells from ferroptosis through non-cell-autonomous functions and activities. In the following sections, we will discuss these metabolic factors and pathways from a cell-autonomous perspective followed by a description of the ways these pathways are circumvented by factors in the TME.

## Intrinsic regulation of ferroptosis

**Figure 2:**
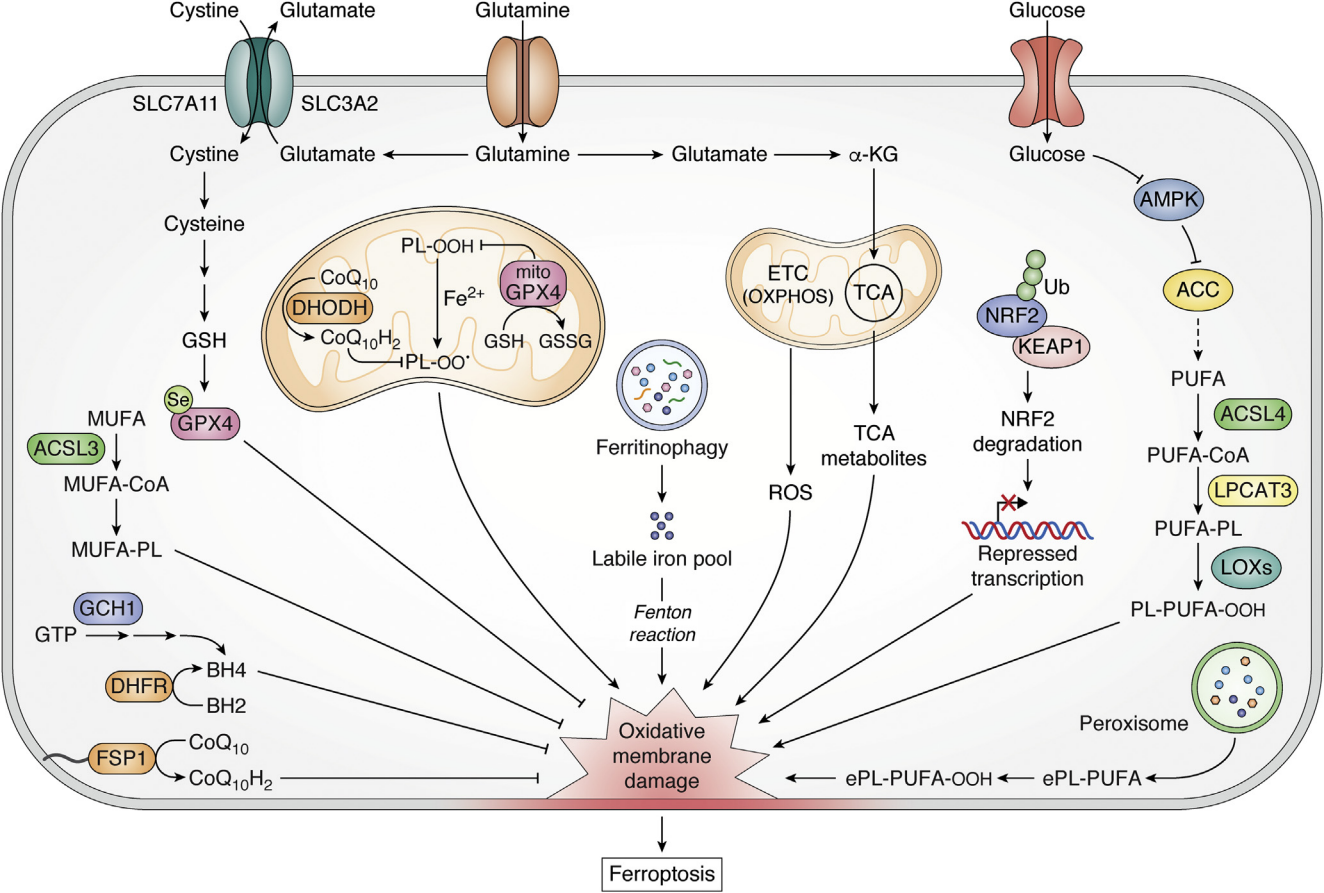
**Additional pathways governing the intrinsic regulation of ferroptosis.** Several cell-autonomous or intrinsic mechanisms modulate cancer cell sensitivity to ferroptosis. This nonexhaustive list includes metabolic pathways that regulate PUFA and MUFA levels, mitochondria respiration and bioenergetics, ferritinophagy, NRF2 antioxidant system, and other direct lipid peroxide neutralizing pathways, *e.g.*, FSP1 and DHODH, which are independent and parallel to GSH-GPX4. αKG, alpha ketoglutarate; ACC, Acetyl-CoA carboxylase; ACSL3, acyl-CoA synthetase long-chain family member 3; ACSL4, acyl-CoA synthetase long-chain family member 4; AMPK, 5′ adenosine monophosphate-activated protein kinase; BH2, dihydrobiopterin; BH4, tetrahydrobiopterin; CoA, coenzyme A; CoQ_10_, Coenzyme Q10; CoQ_10_H_2_, Coenzyme Q10 or ubiquinol; DHODH, dihydroorotate dehydrogenase; DHFR, dihydrofolate reductase; ePL-PUFA, ether phospholipid-polyunsaturated fatty acids; ETC, electron transport chain; FSP1, ferroptosis suppressor protein1; GCH1, GTP cyclohydrolase 1; GPX4, glutathione peroxidase 4; GPX4^mito^, mitochondrial GPX4; GSH, glutathione (reduced); GSSG; glutathione (oxidized); KEAP1, Kelch-like ECH-associated protein 1; LIP, labile iron pool; LOXs, lipoxygenases; LPCAT3, lysophosphatidylcholine acyltransferase 3; MUFA, monounsaturated fatty acid; MUFA-PL, monounsaturated fatty acid phospholipid; NRF2, nuclear factor erythroid 2-related factor 2; OXPHOS, oxidative phosphorylation; PLOO, phospholipid hydroperoxyl radical; PLOOH, phospholipid hydroperoxide; PUFA, polyunsaturated fatty acid; PUFA-PL, polyunsaturated fatty acid phospholipid; ROS, reactive oxygen species; Se, Selenium; TCA, tricarboxylic acid cycle; Ub, Ubiquitin.

The importance and therapeutic potential of ferroptosis in cancer and other diseases have recently received considerable attention. In line with this, so has the list of cell autonomous mechanisms reported to govern ferroptotic sensitivity and resistance (Fig. 2). The section that follows provides a succinct overview of the cell autonomous mechanisms that regulate ferroptosis, as more detailed descriptions have been provided in several excellent reviews (10, 15, 26, 27). Notably, we highlight the literature examples that form the basis of the well-appreciated mechanisms regulating ferroptosis in order to prepare the reader for our more detailed discussion of the non-cell-autonomous mechanisms that follow.

### ACSL4 and LPCAT3 axis

Two of the earliest characterized genes in the regulation of GPX4-inhibition mediated ferroptosis are the lipid metabolic genes: acyl-coenzyme A synthetase long-chain family member 4 (*ACSL4*) and lysophosphatidylcholine acyltransferase 3 (*LPCAT3*) (16, 28, 29, 30). *ACSL4* and *LPCAT3* were found to be critical proferroptotic genes, which encode proteins that remodel the cellular membrane lipid architecture to execute ferroptosis. The protein encoded by *ACSL4* functions in the acylation of long-chain fatty acids, a step that is crucial for the biosynthesis of long-chain PUFA-CoA, including arachidonic acid-CoA (AA-CoA) and adrenic acid-CoA (AdA-CoA). While the protein encoded by *LPCAT3*, on the other hand, mediates the incorporation of these and other acylated AA into membrane phospholipids (16, 28, 29, 30).

*ACSL4* expression directly correlates with sensitivity of cancer cells to ferroptosis; deletion of *ACSL4* suppresses ferroptosis sensitivity, while overexpression sensitizes cells to ferroptosis. Accordingly, *ACSL4* transcript levels were found to be downregulated in ferroptosis-resistant cancer cell lines (29). Deletion of *ACSL4* and *LPCAT3* prevented the integration of PUFA into the membrane bilayer, thereby depleting the substrate for oxidative lipid damage (30). Remarkably cells with *ACSL4* and *GPX4* codeleted maintained their viability (29), indicating the interconnection between the two enzymes. Hence, the membrane remodeling enzymes are key regulatory nodes of ferroptosis susceptibility.

### PUFAs and MUFAs

PUFAs are the site of oxidative lipid damage and are required for the execution of ferroptosis (31). The abundance of PUFAs determines the extent of available lipid peroxidation sites and thus ferroptosis susceptibility. Moreover, high concentration of PUFAs on lipid membranes has been shown to be positively correlated with increased dependency on GPX4 and enhanced sensitivity to iron-dependent oxidative lipid damage (32). Exogenous supplementation with long chain PUFAs enhances ferroptosis sensitivity, even under conditions of *ACSL4* deletion (28). Additionally, exogenous PUFA in the form of DGLA (dihomo-γ-linolenic acid) was recently described as a metabolic inducer of ferroptosis in human cancer cells, and this could be blocked by ferrostatin-1 or endogenous ether lipids (33). The proferroptotic activity of DGLA appeared to be unique to this class of PUFA, as a similar phenotype was not shared by other PUFAs such as AA, eicosapentaenoic acid (EPA). and docosahexaenoic acid (DHA) (33).

Exogenous monounsaturated fatty acids (MUFAs), such as oleic acid (OA, C18:1), display the opposite effect(s) of PUFAs by potently suppressing ferroptosis. Mechanistically, this involves the competitive decrease of PUFA incorporation into membranes, which blocks the propagation of lipid ROS at the plasma membrane (31, 34). This lipid peroxidation-suppressing and ferroptosis-inhibitory role of MUFA is dependent on the activity of the protein encoded by the acyl-coenzyme A synthetase long-chain family member 3 (*ACSL3*) gene, which allows for the selective incorporation of MUFAs into membrane lipids. Treating cells with exogenous MUFAs decreased cellular PUFA-PLs levels, ultimately leading to ferroptosis resistance. Accordingly, low expression of *ACSL3* was correlated with increased ferroptosis sensitivity (34). Therefore, the differential incorporation of PUFAs or MUFAs into membranes increases or decreases sensitivity to ferroptosis, respectively.

### FSP1, CoQ_10_, and NADPH

The ferroptosis suppressor protein 1 (FSP1) was recently characterized as a novel lipid peroxidation neutralizing pathway distinct and parallel to the GPX4 pathway. *FSP1* was identified in genetic screens from two independent research groups that sought to determine pathways that govern resistance to GPX4 inhibition-induced ferroptosis in cancer lines. It was discovered that FSP1 provided resistance to ferroptosis under conditions of *GPX4* deletion (35, 36). Specifically, FSP1 is recruited to the plasma membrane where it uses NAD(P)H to reduce coenzyme Q10 (CoQ_10_) to its radical trapping antioxidant form, ubiquinol (CoQ_10_-H_2_). This then blocks oxidative phospholipid damage and directly mitigates lipid peroxidation. Deletion of *FSP1* in cancer cells results in increased lipid ROS even in the presence of functional GPX4, and overexpression of *FSP1* blocks propagation of lipid ROS in RSL3-treated cells. These collective results illustrate that FSP1 serves as a complementary pathway to GPX4 to block lipid peroxidation (Fig. 2).

### BH4 and DHFR

A recent metabolism-centered genetic screen identified a novel role of the cofactor tetrahydrobiopterin (BH4) and the enzyme dihydrofolate reductase (DHFR) in the protection against ferroptosis (37). BH4 was found to be a potent endogenous radical-trapping antioxidant that regulates sensitivity to ferroptosis induced by GPX4-inhibition, but not cysteine depletion. The radical-trapping antioxidant role of BH4 was distinct from its function as a cofactor to enzymes involved in hydroxylation of aromatic amino acids. The biosynthesis of BH4 from GTP involves several steps catalyzed by the rate-limiting enzyme, GTP cyclohydrolase 1 (GCH1). Genetic deletion of *GCH1* reduced intracellular levels of BH4 and decreased the antioxidant capacity of the cells. These BH4-deficient cells showed enhanced sensitization to GPX4 inhibition-induced ferroptosis. Consistently, treating BH4-deficient cells with exogenous BH4 in the form of dihydrobiopterin (BH2), the dehydrogenated product of BH4, protected against and rescued RLS3 and ML162-induced ferroptosis, but not Erastin-induced ferroptosis. In this setting, DHFR was involved in the restoration of BH4 from BH2 (37) (Fig. 2).

### PUFA-ether phospholipids (PUFA-ePLs)

Contrasting results have been presented on the role of PUFA-ether phospholipids (PUFA-ePLs) in ferroptosis regulation, thus suggesting the involvement of various cellular and genetic contexts in ferroptosis. Perez *et al.* (33) showed that ether lipids resist the induction of lipid peroxidation and ferroptosis induced by exogenous dihomo-γ-linolenic acid (DGLA) in *Caenorhabditis elegans* and human cancer cells (33). This observation was consistent with the role of endogenous ether lipids as antioxidants that protect PUFAs from lipid peroxidation (38, 39). In contrast, Zou *et al.* (40) found that PUFA-ePLs, synthesized by peroxisomes, have a proferroptotic function. In this study (40), it was revealed that PUFA-ePLs served as substrates for lipid ROS propagation and sensitized cells to ferroptosis (40). Whole-genome CRISPR-Cas9 screens with GPX4 inhibitors identified several peroxisomal genes (*e.g.*, *PEX3*, *PEX10*) and ether-lipid biosynthesis genes (*e.g.*, *AGPS*, *FAR1*), which when deleted, promoted resistance to ferroptosis induced by GPX4-inhibition (40). Thus, this work suggests that PUFA-ePLs synthesized by peroxisomes could represent a vulnerability wherein cells downregulate PUFA-ePLs as a strategy to evade ferroptosis and upregulate PUFA-ePLs as substrates of lipid ROS to promote sensitivity to ferroptosis (40) (Fig. 2). Further investigation will be required to shed more light on the role of ether lipids in ferroptosis susceptibility.

### Autophagy and ferritinophagy

Autophagy is an evolutionarily conserved cellular degradation pathway (41). Selective forms of autophagy exist in which discrete subcellular compartments can be targeted for degradation. Relevant here is the regulation of iron availability by ferritinophagy—an autophagy-mediated degradation of iron-bound ferritin (42). In ferritinophagy, NCOA4 is the selective cargo receptor mediating autophagic degradation of ferritin to release iron into the intracellular labile iron pools (LIP) (43, 44). The availability of LIP increases the iron-dependent Fenton lipid peroxidation reactions that trigger ferroptosis (Fig. 2). Reciprocally, increases in ferritin hinder ferroptosis by decreasing the LIP, while ferritinophagy sensitizes cells to ferroptosis *via* the NCOA4-mediated degradation of ferritin. In mouse embryonic fibroblasts (MEF) (45, 46), human pancreatic cancer cells (46), and human sarcoma cells (45, 46), genetic or pharmacological inhibition of autophagy impaired Erastin-induced accumulation of lipid ROS and ferroptosis. On the other hand, increased autophagic flux was associated with enhanced Erastin- and cysteine deprivation-induced tumor ferroptosis (45, 46). Mechanistically, overexpression of *NCOA4* diminished ferritin levels, increased cellular LIP, and consequently enhanced Erastin-induced ferroptosis. Conversely, the knockdown of *NCOA4* increased levels of ferritin, decreased labile iron, and diminished sensitivity of cancer cells to Erastin-induced ferroptosis (46). Furthermore, NCOA4-mediated ferritinophagy is modulated by the metabolic state of the cell. For example, inhibition of glutamate oxaloacetate transaminase 1 (GOT1) in pancreatic cancer cells induced energetic stress, marked by the induction of autophagy and ferritinophagy. This led to increased intracellular LIP and sensitized pancreatic cancer cells to various inducers of ferroptosis (47). The regulation of ferroptosis by ferritinophagy is, therefore, *via* modulating the availability of cellular labile iron for lipid peroxidation reactions.

### NRF2 regulation

The transcription factor nuclear factor erythroid 2-related factor 2 (NRF2) is a master regulator of redox homeostasis and signaling, which modulates the cellular antioxidant mechanisms in addition to other functions in xenobiotic metabolism and cell proliferation. Under homeostatic conditions, levels of NRF2 are kept comparatively low by the activity of E3-ubiquitin ligase complexes, including Kelch-like ECH-associated protein 1-Cullin 3 Ring box 1 (KEAP1-CUL3-RBX1) (48). However, when cells are exposed to oxidant stressors or under conditions of gene alterations, chemical inhibition, or disruption of binding of the ligase complexes to NRF2 protein, NRF2 is released from its interaction with the ligase complex, is stabilized, and is translocated into the nucleus where it activates transcription of antioxidant genes. Several proteins and enzymes that impinge on ferroptosis are encoded by NRF2 target genes, these include proteins involved in iron homeostasis (ferritin, ferroportin, heme-oxygenase) (49, 50) and antioxidant functions (system x_C_^−^, glutathione synthase, GPX4) (48, 50, 51).

NRF2 is upregulated in several cancer types where it serves a tumor-promoting function and confers poor prognosis (50, 52, 53). Consistent with its antioxidant role and upregulation of ferroptosis target genes, NRF2 generally promotes ferroptosis resistance, while genetic silencing or pharmacological inhibition of its activity increases sensitivity to ferroptosis agents (50, 52, 54) (Fig. 2). The hyperactivation of NRF2 was revealed to be critical to the growth of non-small-cell lung cancer (NSCLC) spheroids as it suppressed the induction of ferroptosis in the core of the cancer spheroids (51, 55). Importantly, the combination of GPX4 inhibition and NRF2 silencing efficiently eradicated the tumor cells within spheroids, highlighting the potential for dual targeting of NRF2 and ferroptosis in solid tumors.

### Mitochondria

The first indication that mitochondria were involved in ferroptosis came from evidence of alterations in their morphology in ferroptotic cancer cells, including smaller size, enlarged cristae, and increased membrane density (6, 28, 56). Yet, Erastin treatment effectively induced ferroptosis in mitochondria-DNA depleted cancer cells that were unable to respire (6). Moreover, mitochondria are not essential for the ferroptotic inhibitory function of ferrostatin-1 (18). On the other hand, studies have linked mitochondrial glutamine metabolism to ferroptosis, as glutaminolysis promoted serum-dependent oxidative cell damage and ferroptosis under conditions of either full amino acid or cysteine deprivation (57).

The role of mitochondria was investigated by Gao *et al.* (58) in a study that implicated the mitochondria in cysteine deprivation-induced ferroptosis. Targeting the mitochondria diminished cysteine deprivation, but not GPX4 inhibition-induced, ferroptosis (58). Metabolite intermediates of mitochondrial tricarboxylic acid cycle (TCA) pathway such as α-ketoglutarate, fumarate, succinate, malate, and the components of the electron transport chain (ETC) were revealed to modulate sensitivity of cells to cysteine deprivation-induced ferroptosis. Impairment of the TCA cycle or ETC diminished sensitivity to cysteine deprivation or Erastin treatment (Fig. 2). Interestingly, the loss of function of fumarate hydratase (*FH*), a tumor suppressor gene in renal cancer, was found to promote ferroptosis resistance. This was posited to be an underlying mechanism of its tumor suppressive function, wherein the loss of function mutation in *FH* results in tumor growth benefits.

In another study (59), a genome-wide CRISPR screen in tandem with small-molecule mitochondrial inhibitors revealed a synthetic lethal interaction between *GPX4* deletion and mitochondrial dysfunction. The loss of *GPX4* enhanced the toxic effects of mitochondrial inhibitors and sensitized cancer cells to ferroptosis. Indeed, the synthetic lethal interaction and ferroptosis phenotype were reversed when mitochondrial specific *GPX4* was overexpressed in the *GPX4* KO cells (59). Moreover, *GPX4* expression was shown to be induced in response to mitochondrial dysfunction *in vitro* and *in vivo*. This study (59) supported a potential role of mitochondria as an important site of ferroptosis regulation. Similarly, the inner mitochondria membrane-localized enzyme, dihydroorotate dehydrogenase (DHODH), was shown to modulate ferroptosis sensitivity wherein the deletion or inhibition of the enzyme promoted or sensitized cancer cells to ferroptosis (60). Interestingly, the combination of *GPX4* silencing and *DHODH* deletion, but not either alone, induced robust mitochondrial lipid peroxidation, which could be blocked by mitochondrial specific radical trapping antioxidants (60). To reconcile some of the contrasting findings on the role of mitochondria in ferroptosis, it is worth noting that there are differences in the model of mitochondria elimination in the different studies. These included mitochondria DNA deletion (6) *versus* eradication of the entire organelle (58) *versus* small-molecule inhibition of the ETC (59). Altogether, the context-dependent roles of the mitochondria and mitochondrial metabolism will require future studies.

### Energy metabolism and AMPK

As discussed above, cellular energy metabolism has been linked to ferroptosis susceptibility. Recent work from Lee *et al.* (61, 62) revealed that energetic stress induced by depletion of intracellular glucose or ATP protected cells from ferroptosis. AMPK is a sensor of the cellular bioenergetic state, which is activated upon energetic stress resulting from an increase in AMP/ATP ratio. The phosphorylation-induced activation of AMPK leads to the inhibition of ATP-utilizing anabolic biosynthetic pathways and the activation of ATP-generating catabolic pathways. In MEFs, glucose starvation, subsequent ATP depletion, and activation of AMPK were found to mitigate lipid peroxidation and protect cells from ferroptosis induced by classic ferroptosis inducers (*i.e*., cystine depletion, Erastin, RSL3). In a spectrum of cancer cell lines, high AMPK levels correlated with increased ferroptosis resistance. Moreover, the molecular mechanism of ferroptosis blockade by AMPK activation did not involve mTORC activation, regulation of iron levels, or autophagy. However, the mechanism was mediated in part by the AMPK phosphorylation and inactivation of downstream acetyl-CoA carboxylase (ACC). ACC is a rate-limiting enzyme in the fatty acid biosynthetic pathway, and its phosphorylation inhibits fatty acid synthesis. In sum, the activation of ACC by AMPK led to the decreased generation of PUFAs, the substrate that drives ferroptosis.

In a related study (63), Song *et al.* (63) found that inhibition of pyruvate dehydrogenase kinase 4 (PDK4) promoted cysteine depletion-induced ferroptosis in pancreatic cancer cells. Through siRNA screening, *PDK4* was identified as a key gene that mediated metabolic resistance to ferroptosis. PDK4 acted by blocking pyruvate entry into the TCA cycle, thereby diminishing fatty acid biosynthesis and PUFA production, in a manner akin to that described for activation of AMPK.

## Extrinsic regulation of ferroptosis

**Figure 3:**
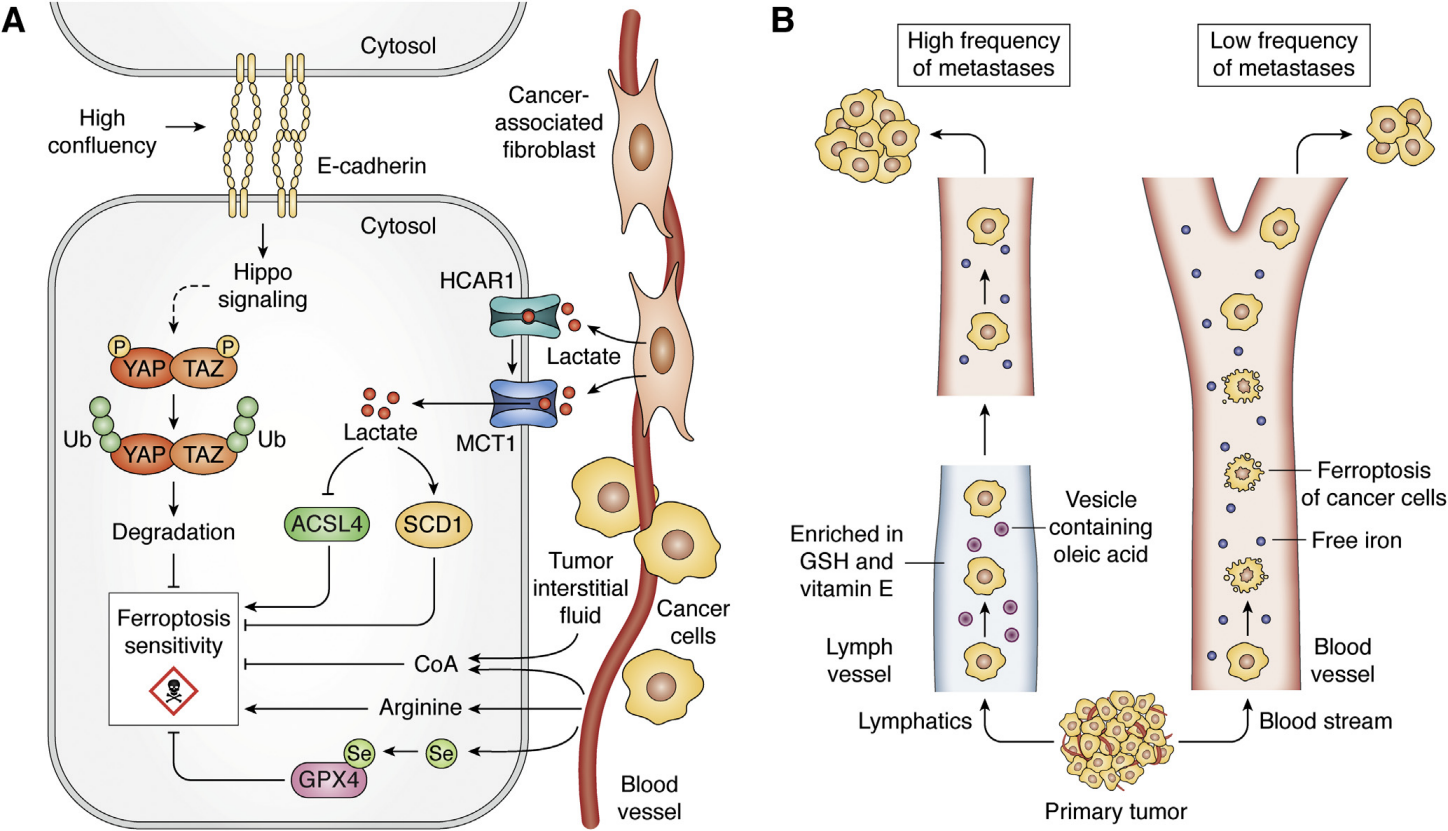
**Extrinsic regulation of ferroptosis.***A*, E-cadherin/hippo signaling and the downstream YAP/TAZ pathways mediate ferroptosis insensitivity with increased cell contact; MCT1-mediated uptake of lactate (*red dots*) regulates ferroptosis *via* ACSL4 and SCD1; availability of Arginine promotes sensitivty to ferroptosis, while CoA diminishes sensitivity to ferroptosis; and supplementation with Selenium (Se) enables ferroptosis resistance through GPX4. *B*, unlike blood, which contains greater levels of free iron, the lymph provides a reducing and antioxidant-rich environment with higher levels of GSH, Vit E, and vesicles containing oleic acid, all of which protect metastasizing tumor cells from ferroptosis. ACSL4, acyl-CoA synthetase long-chain family member 4; CAF, cancer associated fibroblasts; CoA, coenzyme A; GSH, Glutathione; HCAR1, Hydroxycarboxylic acid receptor 1; MCT1, monocarboxylate transporter 1; P, Phosphate; SCD1, stearoyl-CoA desaturase enzyme 1; TAZ, transcriptional co-activator with PDZ-binding motif; U, Ubiquitin; Vit E, Vitamin E; YAP, Yes-associated protein 1.

In this section, we distinguish extrinsic regulators of ferroptosis from the intrinsic pathways discussed in the preceding section as those that occur through interaction with other cells or capture of micronutrients from the serum (Fig. 3). These classifications are somewhat arbitrary in nature and are meant to serve as a bridge between the cell-autonomous pathways that regulate ferroptosis and the non-cell-autonomous mechanisms at play in the TME. Furthermore, the intrinsic and extrinsic pathways are not mutually exclusive.

### Cell-cell contact

High cell density, and the resultant increase in cell-to-cell contact, diminishes sensitivity to ferroptosis induced by various strategies (*e.g.*, cysteine depletion, GPX4 inhibition). This process is linked to cell–cell interactions mediated by E-cadherin (64) (Fig. 3*A*).Higher levels of E-cadherin and downstream Hippo pathway signaling negatively regulated ferroptosis across a spectrum of cancer cell lines. YAP and TAZ are two transcriptional coactivators downstream of Hippo signaling regulated by cell contact (65). High cell confluency results in the retention of YAP/TAZ in the cytosol and their targeted degradation by the proteasome. Conversely, low cell density promotes the translocation of YAP/TAZ to the nucleus, which consequently enables the transcriptional activation of genes regulating cellular growth, survival, and migration (65, 66, 67).

In colon cancer cells, the effector molecules downstream of YAP signaling that mediate ferroptosis sensitivity were found to include transferrin receptor (TRFC) and ACSL4. In line with this model, the absence of YAP abolished ferroptosis sensitivity, while increased expression of *TRFC* and *ACSL4* enhanced ferroptosis in confluent cells previously insensitive to ferroptosis (64). In a model of renal cell carcinoma, the molecular mechanism for ferroptosis resistance in highly confluent cells involved TAZ-induced regulation of epithelial membrane protein 1 (EMP-1) and NADPH oxidase 4 (NOX4). In a low-cell-density state, the presence of TAZ increased the expression of EMP1, which subsequently modulated the levels and activity of the lipid ROS regulator, NOX4, driving ferroptosis (68). Taken together, these studies demonstrate that ferroptosis regulation in the context of cell–cell interaction is modulated similarly upstream by Hippo signaling but differently downstream, *via* YAP/TAZ, in a tumor-type-dependent manner (64, 68).

### Serum-derived nutrients

Selenium (Se) is a metal and micronutrient obtained from the blood that is required for the biosynthesis of the amino acid selenocysteine. Selenocysteine is incorporated into a class of proteins known as selenoproteins, which includes GPX4 (Fig. 3*A*) (69). The critical role of selenocysteine for the catalytic activity of GPX4 was demonstrated by replacing the codon for selenocysteine with that of cysteine in the GPX4 active site. Under these circumstances, the mutant GPX4 did not recapitulate the antiferroptotic and cell-survival effects of the wild-type protein. Mechanistically, it was observed that the wild-type protein displayed intrinsic resistance to peroxidation upon exposure to peroxides. In contrast, the cysteine mutant was irreversibly oxidized when exposed to peroxides, and this led to the inactivation of the enzyme and cell death by ferroptosis (70). Moreover, the observations that selenium supplementation upregulated GPX4 expression and enhanced resistance to ferroptosis further corroborate the modulatory role of selenium in ferroptosis (71, 72).

Arginine is a nonessential amino acid that has numerous important roles in metabolism outside of its proteinogenic function, including the regulation of cell growth through mTOR and immune cell function (73, 74). A recent study demonstrated that depletion of arginine repressed Erastin-induced, but not GPX4-induced, ferroptosis across a spectrum of cancer cell lines (75). This is consistent with previous observations that ferroptosis induced by inhibition of system x_C_^−^ or cysteine depletion displays distinct mechanisms from those induced by GPX4 inhibition or its gene deletion (37). Arginine deprivation-induced suppression of ferroptosis occurred in a manner independent of its function as sensor for mTORC1 or the GCN2/ATF4 pathway (75). The precise mechanistic roles remain to be determined, a finding that is of great interest for cancer therapy. For instance, arginine is degraded by various cell types in the TME, in part to limit immune function (74, 76), while the presence of arginine in the TME promotes metabolic fitness and survival of T cells, factors that are important for antitumor responses (77). These findings suggest that the depletion of arginine in the TME may also impair the ability to induce tumor ferroptosis and thereby enable cancer cell persistence.

In a recent study elucidating the cysteine dependency of pancreatic cancer, it was demonstrated that depletion of GSH was necessary but not sufficient to induce ferroptosis (78). The authors then used mass-spectrometry-based metabolomics profiling of heavy carbon (^13^C)-labeled cystine to determine other fates of cysteine (78). This demonstrated a rapid accumulation of cystine-derived carbon into coenzyme A (CoA). Under conditions of system x_C_^−^ inhibition, exogenous supplementation of CoA was able to rescue ferroptosis in pancreatic cancer cells. Furthermore, combined inhibition of coenzyme A and GSH biosynthesis potently induced ferroptosis, where each agent alone had modest impact (78). Interestingly, CoA has also been implicated in the ferroptosis resistance seen in MEFs harboring the African specific S47 variant of the p53 tumor suppressor gene (79). Together, these results extend the role of cysteine metabolism beyond GSH and raise interesting questions about the role of CoA in ferroptosis. CoA is also present in the tumor interstitial fluid of pancreatic tumors (80), and recent work has described how it can enter cells (Fig. 3*A*) (81). However, its downstream functions in ferroptosis remain to be determined but may include inputs into saturated fatty acid and/or sterol (*i.e.*, CoQ10) biosynthesis (78).

Lactate is an important component of the tumor milieu (82). In hepatocellular carcinoma, lactate was demonstrated to be a negative regulator of ferroptosis. Both exogenous lactate and cancer-associated fibroblasts (CAFs)-conditioned media enriched in lactate were shown to rescue Erastin- and RSL3-induced lipid peroxidation and ferroptosis (83). The blockade of lactate uptake and signaling, *via* genetic silencing or with the pharmacological agent, AZD3965, enhanced lipid ROS and ferroptosis *in vitro* and in tumor-bearing immunodeficient mice (83). Mechanistically, high lactate levels upregulated the pyruvate/lactate transporter monocarboxylate transporter 1 (MCT1) and the lactate receptor hydroxycarboxylic acid receptor 1 (HCAR1). The elevated MCT1 resulted in the downregulation of ACSL4, and upregulation of stearoyl-CoA desaturase enzyme 1 (SCD1)—an enzyme critical for MUFA synthesis (83) (Fig. 3*A*). Thus, the effects of lactate occur *via* perturbation of lipid metabolism products, PUFAs and MUFAs, which modulate ferroptosis sensitivity.

### Lymphatic system

Beyond circulating metabolites and intercellular communication, recent evidence implicates the lymphatic system in providing an antiferroptotic environment for metastasizing tumor cells (84). In contrast to the blood, the lymph was found to be enriched with higher levels of GSH, vitamin E, and lower amounts of free iron. This reducing and antioxidant-rich environment of the lymph inhibited oxidative stress and lipid ROS production, prevented ferroptosis of the trafficking melanoma cells, and thus promoted tumor survival and metastasis to lymph nodes and distal sites. Mechanistically, oleic acid (a ferroptosis-inhibitory MUFA) was found to be present at higher levels in the lymph compared with blood and was a key determinant of the survival of melanoma cells trafficking through the lymph. The origin of the oleic acid in the lymph, and the attendant protective effects, was mechanistically linked to triacyl-glycerides (TAGs) trafficked within ApoB+ vesicles (Fig. 3*B*). Additionally, expression of *ACSL3*, which facilitates incorporation of MUFAs such as oleic acid into membranes, was found to be essential for promoting metastasis (84). Altogether, this study (84) illuminated how the unique milieu of the lymph protects tumor cells from ferroptosis and thereby allows and promotes tumor metastasis.

## Ferroptosis and tumor immunity

The role of the TME in regulating ferroptosis, especially in the context of tumor immunity, is an area of considerable recent interest and therapeutic potential. This is because the nature and properties of the heterogenous immune cells in the TME shape tumor progression, metastasis, and response to therapy by altering the balance between protumor and antitumor immune responses (85). Moreover, how the cells in the TME are influenced by ferroptosis, or mediate ferroptosis sensitivity, determines the efficacy of ferroptosis induction *in vivo*. This understanding is crucial for the development and translation of ferroptosis therapeutics, including rational combination with immunotherapy. In the section that follows, we highlight recent examples from the literature on the role of ferroptosis in tumor immunity, contextualize the emergent data, and discuss their limitations. Of note, many of the *in vivo* studies examining ferroptosis to date have been performed in immune-deficient xenograft models. These transplant models lack the native architecture of a tumor, including the complex stroma, as well as *bona fide* immune system. This made it impossible to study the impact of antitumor immunity, which some studies have indicated both act through and respond to ferroptosis (86, 87). Reciprocally, data from other models have also suggested that ferroptotic cells release danger signals that could enable evasion of immune surveillance (88, 89). The influence of these factors is not accurately considered in the absence of a complete immune system, and where such limitations are evident, these will again be noted and discussed.

### Release of DAMPs

**Figure 4:**
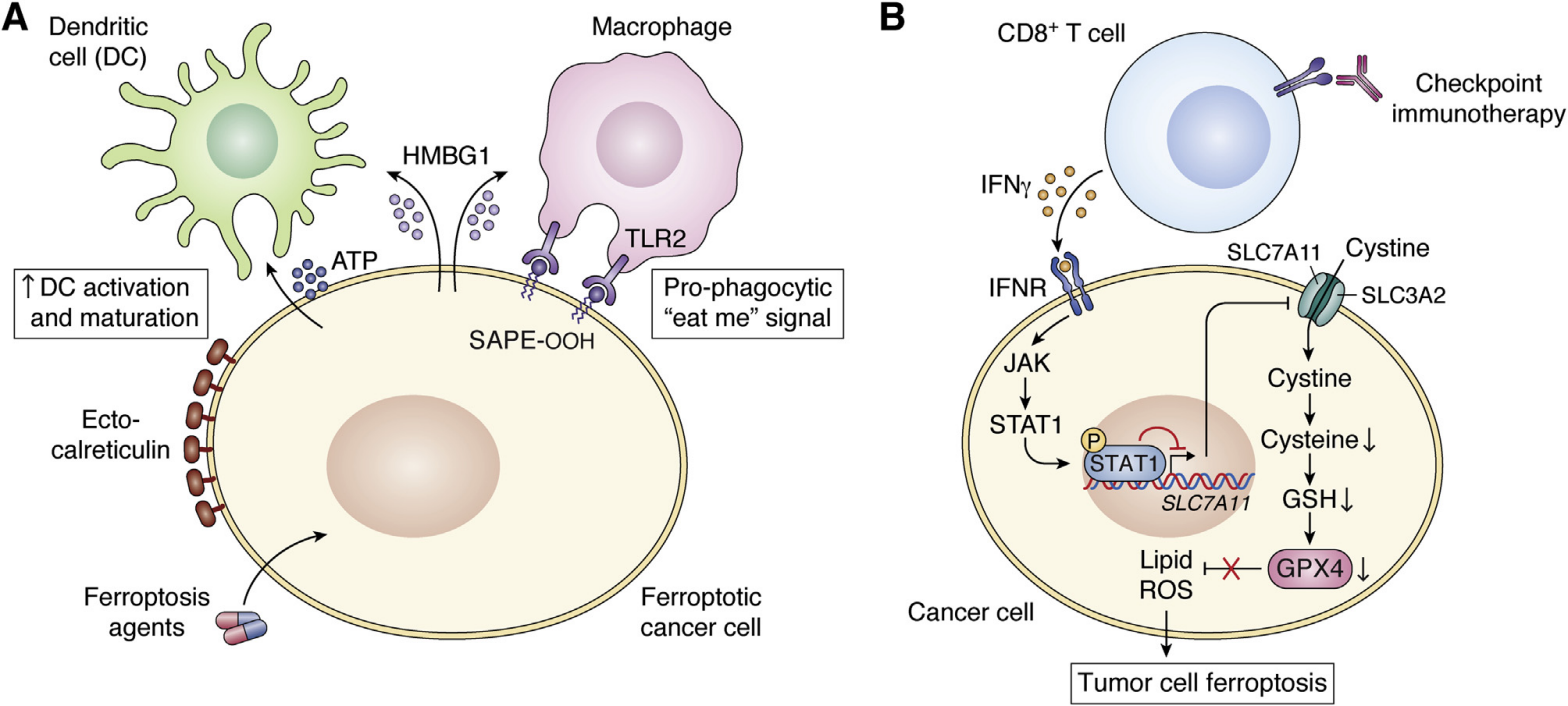
**Antitumor roles of ferroptosis in the tumor immune microenvironment.***A*, immune-stimulatory effects of ferroptosis. Ferroptosis agents induce the release of immunogenic damage associated molecular patterns (DAMPs) such as ATP, HMGB1, and exposure of calreticulin on cell surface (ecto-CRT) from cancer cells. Early- but not late-stage released/exposed DAMPs result in the activation and maturation of professional antigen presenting cells (APCs), such as dendritic cells and macrophages, leading to activation of cytotoxic T cells. In addition, ferroptotic cells release/expose SAPE-OOH, an oxidized phospholipid molecule, which serves as a pro-phagocytic or “eat-me” signal by binding to its cognate receptor, TLR2, on macrophages. *B*, immune-checkpoint activated CD8^+^ T cell-mediated ferroptosis of tumor cells. Activated CD8^+^ T cells secrete IFNγ, which binds to interferon receptor on tumor cells activating the JAK/STAT pathway and causing the translocation of phosphorylated STAT1 into the nucleus where it represses transcription of *SLC7A11*. The decreased level of the cystine importer leads to cysteine depletion, diminished GSH levels, and impaired GPX4 activity. Loss of GPX4 activity eventually results in the accumulation of lipid ROS and tumor ferroptosis. ATP, adenosine triphosphate; ecto-CRT, cell-surface calreticulin; GPX4, Glutathione peroxidase 4; GSH, Glutathione; HMGB1, high mobility group box protein 1; IFNγ, interferon gamma; IFNR, interferon receptor, JAK, Janus kinase; P, Phosphate; ROS, reactive oxygen species; SAPE-OOH, oxidized phospholipid, 1-steaoryl-2-15-HpETE-sn-glycero-3-phosphatidylethanolamine; STAT1, signal transducer and activator of transcription 1; TLR2, toll-like receptor 2.

Damage-associated molecular patterns (DAMPs) are endogenous molecules released or exposed by dying cells and in response to tissue damage. Such factors include ATP, high-mobility group box protein 1 (HMGB1), cell-surface calreticulin, and interferon alpha/beta (IFNα/β). These DAMPs act as chemoattractants or adjuvants that activate innate immune cells such as professional antigen presenting cells—macrophages and dendritic cells (90), which then go on to activate CD4 T and CD8 T cells of the adaptive immune system. Early evidence of DAMP release from ferroptotic cells indicated that MEFs and cancer cells released HMGB1 in a mechanism dependent on the activation of the autophagic machinery (91). The HMGB1 released by ferroptotic cancer cells activated innate immune cells and triggered an inflammatory response that was dependent on HMGB1 binding to the cognate receptor, advanced glycosylation end product-specific receptor (AGER), and not toll-like receptor 4 (TLR4) on macrophages (Fig. 4*A*).

Oxidized phospholipids have recently been classified as DAMPs (92, 93), and these are uniquely enriched in ferroptotic cells. A novel role for a ferroptosis specific-oxidized phospholipid DAMP, cell-surface oxidized phospholipid, 1-steaoryl-2-15-HpETE sn-glycero-3-phosphatidylethanolamine (SAPE-OOH), was shown to mediate cell clearance by macrophages. Cell-surface SAPE-OOH served as an “eat me” signal that allowed the recognition and phagocytic uptake of ferroptotic cells by macrophages (94). This process occurred *via* binding of SAPE-OOH to its identified cognate receptor, toll-like receptor 2 (TLR2), on macrophages (Fig. 4*A*).

### Immunogenicity of ferroptotic tumor cells

Recent evidence has shown that ferroptotic tumor cells can elicit antitumor immune responses, implicating ferroptosis as a potential form of immunogenic cell death (ICD). ICD in cancer therapy is a cell death process characterized by the release of immunogenic DAMPs resulting in a robust and durable activation of the adaptive immune system to eliminate tumor cells (95). Interestingly, RSL3 treatment of fibrosarcoma and glioma cells displayed *in vitro* hallmarks of ICD, which included release of DAMPs, ATP, and HMGB1, phagocytosis of ferroptotic tumor cells by bone-marrow-derived dendritic cells (BMDCs) as well as the maturation of BMDCs (96) (Fig. 4*A*). This immunogenic feature of ferroptotic tumor cells was further confirmed using an immunocompetent *in vivo* prophylactic vaccination model. Herein, subcutaneous vaccination with ferroptotic tumor cells in the flanks of mice led to a protective adaptive antitumor immune response (96). While these findings provided evidence that ferroptotic tumor cells exhibited features of ICD, the antitumor immune response was observed only with cells that had experienced early ferroptosis (*i.e.*, less than 3 h of drug treatment). In contrast, analysis of late ferroptotic cells *in vitro* and *in vivo* did not yield similar outcomes. Rather, the immunogenic phenotype was lost as the late ferroptotic cells neither induced maturation of dendritic cells *in vitro* nor elicited protective antitumor immunity *in vivo*. This study (96) therefore indicates a temporal-specific immunogenicity of ferroptotic cancer cells predicated on the time of treatment and phase of cell death. This finding is also in marked contrast with the immunogenicity induced by apoptosis-inducing ICD agents, which can be maintained from early-stage through late-stage apoptosis (97). This observation therefore sets the stage for more rigorous investigations to define the contextual features for immunogenicity of ferroptosis.

### CD8 T-cell-mediated ferroptosis

It has been demonstrated that CD8 T cells mediate direct tumor cell killing through the induction of ferroptosis of tumor cells. This pioneering work adds to a growing body of mechanisms by which T cells can kill cancer cells, and the therapeutic potential is expounded upon in the section that follows. Mechanistically, the authors (86) showed that activated CD8 T cells released the effector cytokine, interferon gamma (IFNγ), which upon binding to cancer cells triggered the downregulation of *SLC7A11* expression, thereby impairing cystine uptake and resulting in lipid peroxidation and tumor ferroptosis (86) (Fig. 4*B*). This activity could be potentiated by depleting cystine/cysteine levels in the TME *via* an engineered degrading enzyme, Cyst(e)inase, the effects of which were further enhanced when combined with checkpoint immunotherapy. Of particular relevance to clinical data, the authors (86) found that the expression of *SLC7A11* was inversely correlated with the frequency of tumor infiltrating CD8 T cells in human melanoma tumor tissues and that the ferroptosis signature was positively correlated with CD8 T cell effector capacity. Although this remarkable observation was made in the context of immunotherapy-activated CD8 T cells, it raises the possibility of ferroptosis being an intrinsic antitumor CD8 T cell pathway, analogous to traditional pathways such as the granzyme-mediated killing of tumor cells. In addition, it further highlights a potential role for ferroptosis in T-cell-mediated immune surveillance under physiological settings. Indeed, these exciting, albeit hypothetical, concepts warrant extensive investigation.

### Oxidized lipids and T cell ferroptosis

**Figure 5:**
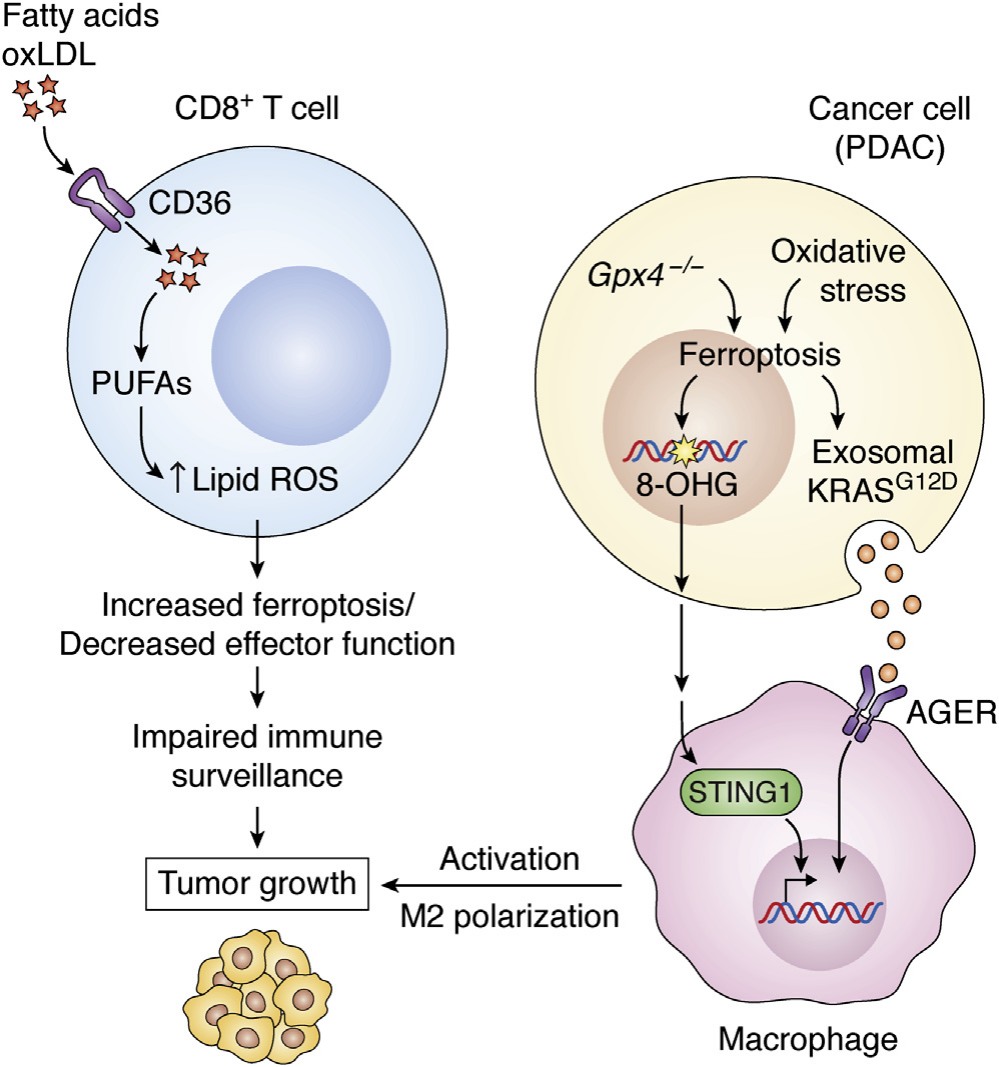
**Protumor roles of ferroptosis in the tumor microenvironment.** The accumulation of lipids, including cholesterol, fatty acids, or oxLDLs, in the TME induces the upregulation of CD36 on CD8^+^ T cells, which leads to further increased uptake of fatty acids and oxLDLs. Elevated fatty acid uptake by CD36 results in higher levels of PUFAs, increased lipid ROS, and enhanced ferroptosis. Ferroptosis of CD8^+^ T cells decreases effector function, impairs immune surveillance, and promotes tumor growth. Moreover, *Gpx4* deletion or high-iron diet promotes pancreatic tumorigenesis *via* the release of oxidized nucleobases, *e.g.*, 8-OHG, which activates the STING pathway resulting in increased macrophage activation and infiltration and tumor growth. Similarly, oxidative stress in pancreatic cancer cells results in autophagy-induced ferroptosis and the release of KRAS^G12D^ packaged in exosomes. Binding of exosomes containing KRAS^G12D^ to AGER receptors on macrophages induced polarization of macrophages to “M2” phenotype and increased tumorigenesis. 8-OHG, 8-hyroxyguanosine; AGER, advanced glycosylation end-product specific receptor; CD36, cluster of differentiation 36; Gpx4, glutathione peroxidase 4; oxLDL, oxidized low density lipoprotein; PUFA, polyunsaturated fatty acids; ROS, reactive oxygen species; STING1, stimulator of interferon genes 1.

As discussed above, T-cell-mediated ferroptosis of tumor cells is beneficial in that it can elicit antitumor immunity and enhance the effect of immunotherapy. However, T cells are also sensitive to ferroptosis. Recent evidence has shown that cytotoxic T cells in the TME can accumulate lipid ROS and undergo ferroptosis, leading to decreased T cell effector function and impaired antitumor immunity. For example, high cholesterol in the TME promoted CD8 T cell exhaustion, while decreased levels of cholesterol in the TME restored CD8 T cell effector function (98). Cholesterol accumulation promoted increased levels of the fatty acid importer CD36, which was associated with increased fatty acid uptake, increased lipid peroxidation, and ferroptosis. Reciprocally, genetic deletion of CD36 on T cells rescued their function and blocked ferroptosis, a feature that was conserved in both murine and human CD8T cells (87). In line with these findings, elevated lipids in the TME have been shown to induce CD8 T cell dysfunction marked by increased CD36 expression and increased uptake of oxidized low-density lipoprotein (oxLDL), which resulted in the induction of lipid peroxidation and impaired CD8 T cell function (Fig. 5). Overexpression of GPX4 in CD8 T cells blocked lipid peroxidation, restored CD8 T cell effector cytokine production, and promoted antitumor immune responses *in vivo* (99). Taken together, these observations suggest an interconnection between the inhibition of lipid peroxidation in maintaining functional CD8 T cell responses and the likelihood of diminished CD8 T effector function as a result of ferroptosis induction in the TME.

Indeed, early studies on the role of GPX4 on T cell activity established that GPX4 is critical to antigen-specific CD4 T and CD8 T cells by functioning to, not only prevent T cell-ferroptosis but to also, confer effective protection against viral and parasitic infections (100). Additionally, the accumulation of oxidized lipids in dendritic cells has been shown to diminish the expression of peptide-MHC I complex and impair cross-presentation of exogenous tumor antigen (101). Moreover, oxidized lipids generated *via* the activity of lipid oxidizing enzyme 12/15-LOX have been found to inhibit the maturation of dendritic cells *via* the activation of NRF2 (84). Thus, it is conceivable that increased ferroptosis, accompanied by release of oxidized lipids, could impair DC cross-presentation and CD8 T cell priming in the TME. These observations raise the possibility that systemic GPX4 inhibition could yield confounding or adverse effects on T-cell-mediated antitumor immunity.

### Protumor roles for ferroptosis

Contrasting the previous studies, ferroptosis has also been shown to support tumor growth in certain contexts (Fig. 5). For example, tumor-associated neutrophils were reported to induce lipid peroxidation and iron-dependent tumor cell death in glioblastoma multiforme (GBM) *in vivo* (102). This led to increased tumor killing, yet it also increased aggressiveness of the disease. The mechanism for ferroptotic killing of tumor cells by neutrophils involved intercellular transfer of myeloperoxidase encased in granules to the tumor cells. Reciprocally, overexpression of *GPX4* or silencing of *ACSL4* in the tumors suppressed ferroptosis and diminished disease aggressiveness (102).

Similar roles have been reported for other immune cells in the TEM. For example, in a study of carcinogen-induced intestinal tumor progression in mice, deletion of *Gpx4* in myeloid cells induced the accumulation of ROS and promoted carcinogen-induced intestinal tumor invasion (103). The authors (103) observed increased recruitment of macrophages to the tumor, which released and elevated levels of peroxide in the TME. This resulted in global DNA mutation events in the intestinal cells that promoted tumor progression (103). These findings illustrate how ferroptosis in immune cells can function to promote tumor initiation and disease progression.

Turning our attention to the malignant cells from a pancreatic tumorigenesis model, ferroptosis induction was found to promote tumor formation and progression. The authors (104) employed an autochthonous model of pancreatic tumor initiation in which the *Kras*^*G12D*^ oncogene is expressed in a pancreas-specific manner. Combination with *Gpx4* deletion or the administration of high-iron diet accelerated pancreatic tumorigenesis, as characterized by an elevated stromal response, increased tumor invasion and metastasis, and decreased survival (104). Mechanistically, both *Gpx4* deletion and high-iron diets were found to promote the release of the oxidized nucleoside base, 8-hyroxyguanosine (8-OHG), which subsequently activated the stimulator of interferon genes (STING) pathway. The STING pathway is an innate immune inflammatory response that senses cytosolic DNA leading to a type I interferon-mediated inflammatory response (105). In this *Kras*-driven pancreatic cancer tumorigenesis model, the STING response heightened the infiltration and polarization of macrophages into a protumor phenotype. The protumorigenic phenotypes and decreased survival were rescued by the ferroptosis inhibitor, liproxstatin-1, depleting macrophages, or inhibiting the STING pathway (Fig. 5). In a different study using a pancreatic cancer disease model (106), it was found that ferroptosis promoted autophagy-dependent exosomal release of KRAS^G12D^ protein from pancreatic cancer cells. The extracellular KRAS^G12D^ was bound by and taken up by AGER/RAGE receptors on macrophages and resulted in the tumorigenic polarization of macrophages and promoted pancreatic tumor growth (106).

Collectively, these emerging data on the role of ferroptosis in the TME suggest a dual effect, where ferroptosis is either tumor-promoting or tumor-suppressive, in a cell-type, model, disease state, and cancer-type-dependent manner. Therefore, in order to translate ferroptosis-regulating agents as cancer therapies, there is a need for more rigorous and systematic investigation of ferroptosis that considers the full spectrum of the TME landscape. This will assist in determining conditions under which ferroptosis promotes *versus* inhibits tumor growth *in vivo*. Furthermore, the evidence of a duality of ferroptosis in the TME speaks to the importance of compartmentalizing the induction of ferroptosis to specific cell types.

## Emerging opportunities for targeting ferroptosis in cancer therapy

Employing ferroptosis as a treatment strategy has been hampered by the limited methods to induce and monitor ferroptosis *in vivo*. These drawbacks include the lack of potent, specific drugs with suitable pharmacokinetic and pharmacodynamic profiles, and challenges in defining specific biomarkers for assessing tumor ferroptosis, relative to other forms of tumor cell death, *in vivo*. These shortcomings notwithstanding, several experimental approaches have been developed to test the therapeutic potential of ferroptosis in tumors.

First, genetic strategies have been employed to circumvent the paucity of *in vivo* ready drugs. For example, deletion of the gene encoding xCT has been reported to inhibit tumor growth in both pancreatic cancer xenograft models (107) and established tumors in a *Kras* and *p53*-driven transgenic mouse model of pancreatic cancer (78). In the latter study, Badgley *et al.* developed a seven-allele genetically engineered mouse model of pancreatic cancer in which tumors could be initiated using the Flp-Frt recombination system and whole body *Slc7a11* deletion could be initiated by tamoxifen administration. These animals were monitored for endogenous tumors by ultrasound and then scheduled for a tamoxifen treatment. *Slc7a11* knockout led to inhibition of tumor growth and increased survival in mice (78). Given *Slc7a11* deletion is safe (108), and considering it does not compromise antitumor immune response *in vivo* (109), xCT inhibition is now posed as a promising therapeutic approach for pancreatic cancer.

Second, new generations of pharmacological system x_C_^−^ inhibitors are being developed with better *in vivo* properties. For example, the Erastin analog, imidazole ketone erastin (IKE) is a highly effective and metabolically stable inhibitor of system x_C_^−^. When applied in a diffuse large B cell lymphoma (DLBCL) tumor xenograft model, IKE induced hallmarks of ferroptosis and suppressed growth (110).

Third, a novel, orthogonal strategy for inducing cysteine depletion was recently repurposed to induce and study ferroptosis *in vivo*. Cyst(e)inase is an enzyme engineered to breakdown extracellular cysteine and cystine. Cyst(e)inase exhibits good pharmacokinetic and toxicology profile and remarkable efficacy, depleting circulating cyst(e)ine levels by more than 99% (111). The therapeutic efficacy of Cyst(e)inase has recently been evaluated in a number of *in vivo* tumor models. For example, Cyst(e)inase was shown to induce ferroptosis and suppress tumor growth in human xenograft (112) and endogenous murine tumor models of pancreatic cancer (78), prostate cancer xenografts (111), and breast cancer xenografts (111). Furthermore, it was found that pancreatic cancer cells resistant to Cyst(e)inase treatment upregulated thioredoxin 1 as mechanism of escape. Consequently, treating resistant pancreatic tumor xenografts with auranofin (a thioredoxin reductase inhibitor) sensitized the tumors to Cyst(e)inase treatment and suppressed tumor growth without overt systemic toxicity (112). Interestingly, Cyst(e)inase synergized with radiotherapy to inhibit tumor growth in the B16 melanoma model (113) and also synergized with immune checkpoint therapy (*i.e.*, anti-PDL1) to promote cytotoxic CD8 T cell antitumor immune response in ovarian-tumor-bearing mice (86).

Fourth, beyond evaluating ferroptosis inducers as potential single drug agents in treating cancer, the evidence presented above illustrates that such agents could also serve as effective sensitizers to radiation and immune-based therapies. Although single-agent radiation therapy (RT) has been demonstrated to suppress lung cancer xenografts *via* ferroptosis (114, 115), this effect can be further enhanced in combination with system x_C_^−^ inhibitors, IKE, and sulfasalazine, which in combination strongly suppressed tumor growth in sarcoma xenografts, glioma xenografts, and lung cancer xenografts (114, 115, 116). Further, the genetic deletion of *SLC7A11* in various preclinical tumor models acts synergistically with immune checkpoint immunotherapeutic agents, such as anti-CTLA4, to suppress tumor growth (109).

In addition to classic ferroptosis-inducing agents, certain conventional chemotherapy drugs have been shown to either sensitize cancer cells to ferroptosis, *e.g.*, Statins (117, 118), or to exert their antitumor effects fully or in part *via* ferroptosis, *e.g.*, Cisplatin (119). The combination of radiotherapy and immunotherapy has also been shown to induce ferroptosis in a variety of preclinical tumor models (113). The mode of action of this combinatorial strategy was linked to the suppression of xCT levels and consequent limited cysteine uptake in the tumor cells. This cysteine deprivation was engendered by combined effects of immunotherapy-generated IFNγ and radiation therapy-activated ATM pathway. Ultimately, tumors exposed to radiation and immunotherapy underwent extensive lipid peroxidation and ferroptosis resulting in decreased tumor burden. A summary of ferroptosis agents that have been tested in preclinical models is presented in Table 1. Also included is a synopsis of preclinical testing of the combinatorial application of ferroptosis with other chemotherapeutic agents, radiation therapy, or immunotherapy (Table 2).

**Table 1:** Ferroptosis inducing agents tested *in vivo*, their mechanism of action, and clinical status

Ferroptosis agents	Mechanism	Cancer model	Used in clinic	Ref
Artesunate	Lipid peroxidation	Pancreatic cancer	Yes	(128)
Buthionine Sulfoximine (BSO)	γ-glutamylcysteine synthetase inhibitor	Clear cell renal cell carcinoma	Yes	(129)
Cyst(e)inase	Depletes extracellular cysteine/cystine	Pancreatic, prostate, breast carcinoma	No	(78, 111, 112)
Dihydroartemisinin	Degradation of ferritin, lipid ROS	Lung cancer	Yes	(130)
Imidazole Ketone Erastin (IKE)	System x_C_^−^ inhibitor	Diffuse large B cell lymphoma (DLBCL)	No	(110)
JQ1	Elevated iron, lipid ROS	Lung cancer	Yes	(131)
Piperazine Erastin (PE)	System x_C_^−^ inhibitor	Fibrosarcoma	No	(25)
Sulfasalazine	System x_C_^−^ inhibitor	Head and neck cancer	Yes	(126)
Sorafenib	System x_C_^−^ inhibitor	Hepatocellular carcinoma	Yes	(132)
Withaferin A	Depletion and inactivation of GPX4	Neuroblastoma	No	(133)
Ionizing Radiation (IR)	ROS, lipid ROS, increased ACSL4	Lung cancer	Yes	(114, 115)
Immune checkpoint blockade: anti-PD-L1, anti-CTLA4	IFNγ secretion, xCT suppression, lipid ROS	Melanoma, ovarian cancer	Yes	(86)

Abbreviations: ACSL4, acyl-CoA synthetase long-chain family member 4; CTLA4, cytotoxic T-lymphocyte-associated protein 4; GPX4, glutathione peroxidase 4; GSH, glutathione; IFNγ, interferon gamma, PD-L1, programmed death-ligand 1; ROS, reactive oxygen species.

**Table 2:** Ferroptosis sensitizing combinatorial strategies tested in cancer models *in vivo*

Ferroptosis combinatorial strategies		Mechanism	Cancer model(s)	Ref
Cisplatin	Erastin	Inhibition of GSH synthesis, induction of ferritinophagy	Ovarian cancer	(134)
	Sulfasalazine	GSH depletion, lipid ROS accumulation	Head and Neck Cancer	(135)
BSO	*GOT1* deletion	GSH depletion	Pancreatic cancer	(47)
IKE	Dichloroacetate	System x_C_^−^ and PDK4 inhibition	Pancreatic cancer	(63)
Dihydroartemisinin	*GPX4* deletion	Degradation of ferritin, lipid ROS	Lung cancer	(130)
	Cisplatin	Degradation of GPX4, accumulation of iron	Pancreatic cancer	(136)
Artesunate	*GRP78* deletion	Lipid peroxidation	Pancreatic cancer	(128)
Brequinar	*GPX4* silencing	Lipid peroxidation	Fibrosarcoma	(60)
	Sulfasalazine			
Rapamycin	*GPX4* deletion	Lipid peroxidation	Pancreatic cancer	(137)
Sorafenib	Sigma-1 receptor depletion	Lipid peroxidation, Fe^2+^ elevation	Hepatocellular carcinoma	(138)
Sulfasalazine	Pioglitazone	xCT inhibition and Fe^2+^ elevation	Head and Neck cancer	(126)
	Metformin	Fe^2+^ elevation, lipid ROS	Breast cancer	(139)
	Sorafenib	Ferritinophagy, xCT inhibition	Pancreatic cancer	(140)
	IFNγ	xCT suppression, lipid ROS	Fibrosarcoma	(86)
Oxyfedrine	Sulfasalazine	GSH depletion, 4-HNE accumulation	Colon cancer	(127)
Radiation therapy	IKE, Sorafenib	Lipid peroxidation, decreased GSH	Fibrosarcoma	(116)
	Sulfasalazine	Decreased GSH	Melanoma, Lung Cancer	(114, 115, 141)
	Cyst(e)inase, Anti-CTLA4, Anti- PD-L1	IFN release, xCT suppression, ATM activation, lipid peroxidation	Melanoma	(113)
X-ray irradiation	Erastin	Decreased GPX4 and GSH	Non-small cell lung cancer (NSCLC)	(142)
Immune checkpoint blockade: anti-PD-L1, anti-CTLA4	Cyst(e)inase	xCT suppression, cysteine depletion, lipid ROS	Melanoma	(86)

Abbreviations: 4-HNE, 4-Hydroxynonenal; ATM, ataxia-telangiectasia mutated; BSO, buthionine sulfoximine; CTLA4, cytotoxic T-lymphocyte-associated protein 4; GOT1, glutamate oxaloacetate transaminase 1; GPX4, glutathione peroxidase 4; GSH, glutathione; IFNγ, interferon gamma; IKE, imidazole ketone erastin; PD-L1, programmed death-ligand 1; PDK4, pyruvate dehydrogenase kinase 4; ROS, reactive oxygen species.

Lastly, simple and robust methods to definitively identify ferroptosis *in vivo*, akin to cleaved caspase 3 as a marker for apoptosis, are not currently available. Early studies utilized select pharmacodynamic markers to evaluate the degree of ferroptotic lesions in tumor samples. These markers included the expression of *PTGS2* and *CHAC1*, immunofluorescence, or histological staining of 8-OHdG, MDA adducts, and 4-HNE (25, 78, 110, 120, 121). However, the application of these assays is still limited by the context-dependent utility and the lack of ferroptosis specificity sufficient to distinguish ferroptotic lesions from other cell death programs, oxidative-stress-related damage, or inflammatory conditions. For instance, *PTGS2* is not a highly sensitivity pharmacodynamic marker when tumors are treated with low concentration of system x_C_^−^ inhibitor. Moreover, *PTSG2* can be upregulated in other inflammatory conditions; *CHAC1* is upregulated in system x_C_^−^ inhibition but not GPX4 inhibition; and MDA-adducts can be detected under certain conditions of general oxidative stress not related to ferroptosis (25, 110, 120, 121).

More recent studies have employed combinations of orthogonal approaches and additional biomarkers to more definitively call ferroptotic cell death. A comprehensive analysis of ferroptotic tumor lesions was described by Badgley *et al.*, (78) who examined Cyst(e)inase-treated or autochthonous *Slc7a11*-deleted pancreatic tumors. In this study (78), ferroptosis was classified based on histopathological hallmarks of ferroptosis, such as the accumulation of lipid peroxidation products (4HNE, MDA) and lipid droplet formation with oil red O staining; mitochondrial anomalies and the accumulation of lipid droplet structures by transmission electron microscopy; gene expression changes by RNA sequencing on laser capture micro-dissected tumor sections; and the absence of tumor-specific cleaved caspase 3. This approach, while conclusive, is time- and cost-intensive. In an effort to identify reliable and robust ferroptosis-selective markers without the need for a panoply of orthogonal assays, Feng *et al.* performed a screen for antibodies from mice immunized with membranes from cells undergoing ferroptosis. This led to the identification of 3F3-FMA and its target antigen, transferrin receptor 1 (TfR1), which called ferroptosis in tissue sections across diverse human and mouse models (122). It is expected that future investigations will take advantage of the combination of ferroptosis markers, including anti-TfR1, 3F3-FMA, anti-MDA, anti-4HNE, to establish the role of ferroptosis *in vivo*. The paucity of biomarkers calls for more efforts to identify additional ferroptosis-specific biosignatures for use in preclinical and clinical settings.

## Conclusion and perspectives

Since the term ferroptosis was coined by the Stockwell lab in 2012 (6), numerous studies detailing genetic, transcriptional, and metabolic mediators of this regulated cell death process have emerged. Ferroptosis has been implicated in various pathologies including renal disease, ischemia/reperfusion injury, neurodegenerative diseases, and cancer (123). Whether there are physiological stimuli of ferroptosis and whether ferroptosis play a role in physiological processes such as in tissue development, immune surveillance, or maintenance of cellular homeostasis is largely unknown—a topic requiring considerable attention. Moreover, much of what we know about ferroptosis in the context of cancer biology and cancer therapy has been delineated with cancer cells in culture, conditions that are largely often considered *non*physiologic. Cancer cells in tumors do not exist in isolation as in culture, and their behaviors, properties, and ferroptosis susceptibility are highly influenced by their *in vivo* microenvironment. As an example, it was reported that targeting the ferroptosis gene, *SLC7A11*, in both tumor cells and CAFs, but not in tumors cells alone, decreased tumor growth and metastatic burden in an orthotopic model of human pancreatic ductal adenocarcinoma (PDAC) (124). Indeed, more recent studies are beginning to shed light on the role of non-cell-autonomous regulatory mechanisms, including those from the serum, TME, and immune system. Nevertheless, significant gaps in our knowledge of the impact of ferroptosis in the TME remain with contrasting reports describing a duality of protumor or antitumor effect(s) in a context-dependent manner. Therefore, more rigorous investigations are required to evaluate the impact of tumor ferroptosis on stroma cells such as CAFs, myeloid cells, tumor-associated macrophages, and T lymphocytes, all of which have remained largely underexplored. Such a detailed understanding will be required to harness cell autonomous and non-cell-autonomous ferroptotic mechanisms to design the most effective cancer therapies

Another area of future investigation and of clinical significance is whether metabolic signatures of ferroptosis can find utility as biomarkers and consequently stratify tumor types based on ferroptosis susceptibility. This will be crucial for the application of precision medicine-based approaches that leverage the next generation of ferroptosis agents. In addition, it is pertinent to determine whether the metabolic regulators (or pathways) that predict ferroptosis sensitivity will also provide an adequate therapeutic window. Preclinical studies with Cyst(e)inase (78, 111, 112) and the lack of overt phenotypes in the *Slc7a11* null mouse models (108, 125) suggest that therapeutic window could be readily achievable. Finally, single-agent engagement of ferroptosis will undoubtedly lead to therapeutic resistance, as has been seen in preclinical models (78, 126, 127). Thus, achieving ferroptosis *in vivo* would most likely require combination approaches such as those detailed in Table 2.

## Conflict of interest

C. A. L. has received consulting fees from Astellas Pharmaceuticals and Odyssey Therapeutics and is an inventor on patents pertaining to KRAS-regulated metabolic pathways, redox control pathways in cancer, and targeting the GOT1-pathway as a therapeutic approach.
